# Low Unintended Dural Puncture Rate Using a Flush-Measure-Check-Advance Technique to Perform Combined Spinal-Epidural Anesthesia in Parturients: A Quality Improvement Clinical Series

**DOI:** 10.31486/toj.24.0133

**Published:** 2025

**Authors:** James Riopelle, Julie Gayle, Zubaer Anwar, Jeff Burton

**Affiliations:** ^1^ Department of Anesthesiology, Louisiana State University Health Sciences Center School of Medicine, New Orleans, LA; ^2^ Louisiana State University Health Sciences Center School of Medicine, New Orleans, LA; ^3^ Ochsner Center for Outcomes Research, Ochsner Clinic Foundation, New Orleans, LA

**Keywords:** *Anesthesia–epidural*, *anesthesia–obstetrical*, *anesthesia–spinal*, *post-dural puncture headache*

## Abstract

**Background:**

Reported rates of unintended dural puncture during performance of continuous epidural anesthesia (CEA) or combined spinal-epidural anesthesia (CSEA) have remained steady at approximately 0.5% to 1% since the 1970s.

**Methods:**

A modified method of inserting the Tuohy epidural catheterization needle was used during performance of CSEA in 393 parturients. A single staff/faculty anesthesiologist performed or supervised resident use of a flush-measure-check-advance Tuohy needle insertion algorithm.

**Results:**

The rate of evident Tuohy needle dural puncture during the series was 0%. One parturient experienced a post-dural puncture headache possibly because of intentional subarachnoid puncture with a very small diameter (25 gauge) needle during 2 CSEAs. In 19 parturients, the initial spinal anesthesia portion of CSEA failed, prompting conversion to CEA in 18 parturients and to spinal anesthesia in 1 parturient.

**Conclusion:**

The use of a flush-measure-check-advance Tuohy needle insertion algorithm to reduce the likelihood of unintended dural puncture during performance of CSEA in parturients deserves further study.

## INTRODUCTION

### Advent of Epidural Anesthesia in Obstetric Care

The mid-twentieth century witnessed a breakthrough in the medical care of parturients in labor. The maternal experience changed from one of pain and isolation to one of relative comfort and family bonding. This transformation was largely attributable to the development of continuous epidural anesthesia (CEA): the provision of uninterrupted analgesia (during labor) and/or anesthesia (during cesarean section) by intermittent or continuous injection of a local anesthetic solution through a catheter positioned in the epidural space. To guide the catheter from the skin through subcutaneous tissue and the posterior midline lumbar spinous ligaments and then into the epidural space, special large-bore needles with oval angulated tips were invented, with the Tuohy needle being the most widely used.

### Introduction of Combined Spinal-Epidural Anesthesia

At the beginning of the 21st century, combined spinal-epidural anesthesia (CSEA) began to progressively replace CEA. The principal innovation of CSEA (also a continuous catheter technique) was to initiate a neural blockade by administering 1/10th of the local anesthetic dose required to initiate CEA into the subarachnoid space rather than the epidural space. Such initial spinal dosing provided superior pain relief, was several minutes faster in onset (especially helpful for cesarean section), and greatly reduced the likelihood of the 2 most dangerous complications of large-dose neuraxial local anesthetic bolus administration: (a) total spinal anesthesia (total body paralysis and cardiovascular collapse) resulting from inadvertent injection of the high dose of local anesthetic solution intended for epidural injection into the cerebrospinal fluid and (b) local anesthetic systemic (cardiac) toxicity resulting from the medication bolus entering the bloodstream via a torn epidural vein.

### Unintended Tuohy Needle Dural Puncture

The most common complication of using a Tuohy needle to initiate neuraxial anesthesia is unintended dural puncture. Unintended dural puncture occurs when the needle is overinserted and traverses the dura mater and arachnoid membrane to enter the subarachnoid space. In more than 50% of such cases, continuing drainage of cerebrospinal fluid through the 17-gauge dural tear is followed by the occurrence of a postural post-dural puncture headache, one that is almost always worse when the patient is erect vs recumbent. Such headaches are often severe enough to confine a patient to bed and interfere with maternal-neonatal bonding. Even when effectively treated with an epidural blood patch (a second epidural injection, this time of an aliquot of the patient's own blood), unintended Tuohy needle dural puncture is sometimes followed by the development of chronic headache. Rarely, unintended Tuohy needle dural puncture leads to development of intracranial subdural hematoma,^1^ probably because continued loss of cerebrospinal fluid at the dural puncture site causes caudal traction on cerebral meningeal veins. Of note, successful CSEA, by definition, includes intentional dural puncture with a fine-gauge spinal needle, usually 25 gauge or smaller.

### Project Designed to Reduce the Rate of Unintended Tuohy Needle Dural Puncture

**Figure 1.**
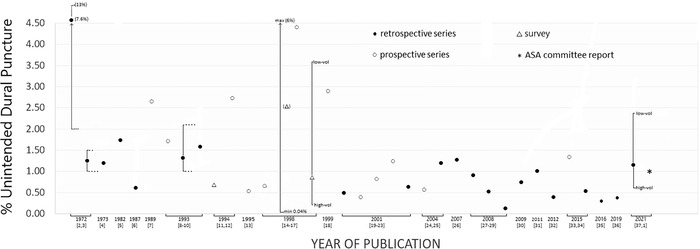
**Published rates of unintended Tuohy needle dural puncture in obstetric patients receiving continuous epidural or combined spinal-epidural anesthesia from 1972 to 2021. Vertical lines show unintended dural puncture rate ranges when the authors reported rates differentiated by performer experience (• novice, •• resident, ••• fellow or registrar, •••• faculty/staff); by facility (max [maximum], rate at the hospital with the highest rate; min [minimum], rate at the hospital with the lowest rate); or by practitioner annual case volume (vol) (high-vol, high caseload volume; low-vol, low caseload volume). Bracketed numbers below publication dates are the articles’ reference list numbers. Open and black circles, open triangles, and the asterisk are series means except for the triangle in parentheses that is from a published series that reported maximum and minimum unintended dural puncture rates but no mean value.** ASA, American Society of Anesthesiologists.

Despite progress in many aspects of CEA and CSEA since the 1970s, the rate of unintended Tuohy needle dural puncture during these procedures has remained stable at approximately 0.5% to 1% (Figure 1).^1-37^

In March 2014, a staff anesthesiologist at a 170-bed acute care suburban hospital began a quality improvement project with the goal of minimizing the rate of unintended Tuohy needle dural puncture in parturients receiving neuraxial anesthesia. The staff anesthesiologist was also a faculty member of an affiliated academic medical center and routinely supervised residents rotating through the hospital's obstetric unit. The anesthesiologist developed a modified, more nuanced algorithm than the traditional binary (+/–) loss of resistance algorithm for Tuohy needle insertion. During the next 72 months, the partly new technique was used in 393 parturients. The project ended in January 2020 when the anesthesiologist was transferred to another hospital that did not offer obstetric care.

## METHODS

The faculty anesthesiologist personally performed or closely supervised performance of neuraxial anesthesia for all patients.

### Combined Spinal-Epidural Kit Used During the Project

The hospital's CSEA tray was an Espocan kit (B. Braun Medical Inc) that included a 90-mm epidural (Tuohy) needle with alternating shiny and matte cm markings and 2 oval orifices: (a) a 17-gauge end orifice (long axis length 2.5 mm) designed to direct an inserted catheter upward from the needle's long axis; and (b) a 25-gauge back-eye orifice (long axis length 2 mm) across from the end orifice to permit transmission of the 25-gauge, 127-mm, pencil-point spinal needle (provided with stylet). The Espocan kit also included a Perifix FX 20-gauge, 92-cm, soft, spring-wound, multiorifice, bullet-tipped epidural catheter, as well as several needles and syringes for diluting and injecting sterile saline and local anesthetic solutions.

### Traditional Technique for Advancing the Tuohy Needle From Skin to the Epidural Space

**Figure 2.**
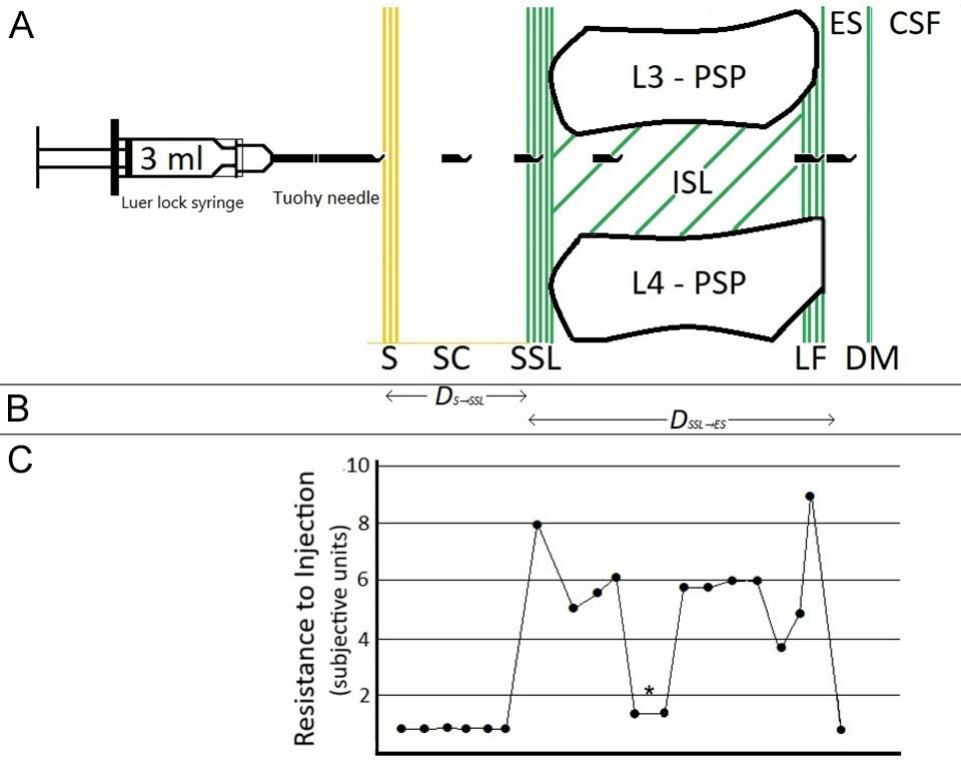
**Resistance to fluid (saline or local anesthetic solution) injection as subjectively measured by the operator inserting a Tuohy needle during performance of combined spinal-epidural anesthesia or continuous epidural anesthesia. (A) Serial Tuohy needle tip and end orifice positions during insertion into the dorsal lumbar midline. (B) Depiction of the distance from the skin to the epidural space (ES) as the sum of the distance from the skin to the supraspinous ligament (SSL) (*D_S→SSL_*) plus the distance from the SSL to the ES (*D_SSL→ES_*). (C) Graph of serial subjective measurements of resistance to fluid injection through the Tuohy needle as the needle tip was advanced from the skin to the ES. A single episode of false-positive loss of resistance is depicted by the asterisk.** CSF, cerebrospinal fluid; DM, dura mater; ES, epidural space; ISL, interspinous ligament; L3-PSP, posterior spinous process of the third lumbar vertebra; L4-PSP, posterior spinous process of the fourth lumbar vertebra; LF, ligamentum flavum; S, skin; SC, subcutaneous layer; SSL, supraspinous ligament.

Traditionally, after injection of a local anesthetic solution into the skin and subcutaneous tissue, a Tuohy needle with stylet is inserted through the skin into the dorsal lumbar midline and then through a gap between adjacent lumbar vertebral posterior spinous processes of any 2 of the lowest 4 posterior lumbar vertebral spinous processes (Figure 2). The stylet is not removed until the needle has been inserted deep enough to have entered interspinous ligament. Tip lodgment in ligamentous tissue is confirmed if the hub end of the needle can maintain its original straight-backward or slightly upward pointing position without support from the operator's hand. The stylet is then removed, and a low-friction 5-mL syringe filled with air or normal saline is attached. With one hand on the syringe and the other hand on the needle, the operator serially advances the Tuohy needle toward the epidural space in 2- to 3-mm increments. After each advance, the operator gently pushes on the syringe plunger and confirms that this incremental advancement has not resulted in any actual injection of air or saline into the patient. Once the end orifice of the Tuohy needle passes through ligamentum flavum (the third ligamentous layer), the same gentle force on the plunger easily injects some or all of the syringe contents into the epidural space. The subjective sensation of reduced resistance to injection of fluid or air as the Tuohy needle tip passes through ligamentum flavum into the epidural space is known as *loss of resistance*. Some operators use the Tuohy needle cm markings to track needle tip insertion depth and begin to make shorter needle advances once they suspect that the Tuohy needle tip has become very close to the epidural space. The usefulness of cm markings for this purpose, however, is limited by the large variability in epidural space depth, principally because of differences in the depth of lower lumbar fatty tissue.

### Technique for Dislodging Tuohy Needle Blockage to Prevent Unintended Dural Puncture

In 2001, Cohen, Maestrado, and Zada reported that their anesthesia residents achieved a 0.5% rate of unintended Tuohy needle dural puncture when providing CEA by disconnecting the low-friction syringe and fully inserting the Tuohy needle stylet once the Tuohy needle tip was perceived to have reached ligamentum flavum.^38^ The purpose was to dislodge any ligamentous tissue plug that might be blocking the needle end orifice. From this point forward, the length of each incremental Tuohy needle advance was reduced to 1 to 2 mm because of the proximity of the needle tip to the epidural space and, just beyond it, the dura mater.^38^

A short while after we adopted the Cohen et al technique, one of our obstetric anesthesiologists modified it to incorporate multiple syringe-needle hub disconnections, stylet reinsertions, and syringe-needle hub reconnections. While this modification appeared to be capable of even further lowering the unintended dural puncture rate, some anesthesiologists considered it to be too inefficient for routine use in patients with normal anatomy.

### Development of a Flush-Measure-Check-Advance Algorithm for Advancing the Tuohy Needle

This quality improvement project was begun with the hope that repeated small—approximately 0.2 mL—flushes of normal saline or dilute local anesthetic solution through the Tuohy needle during insertion would be a more efficient, but equally effective, substitute for multiple syringe-needle hub disconnections and stylet reinsertions. The low-friction, 5-mL, Luer tip syringe supplied in our Espocan CSEA kit proved unsuitable for such use because (a) the force required to inject fluid into ligamentous tissue using a syringe with an internal diameter of approximately 12 mm often exceeded the force a normal person's thumb can exert, and (b) application of force to the syringe plunger that was sufficient to definitely inject fluid into ligamentous tissue usually caused the syringe's Luer tip to disengage from the needle hub, causing the fluid within the syringe to squirt out vigorously. This fluid leak problem was solved by switching to a Luer lock syringe, the tip of which screws into a needle hub. Downsizing to a 3-mL syringe with a 9-mm internal diameter reduced the force needed to inject fluid into ligamentous tissue just enough to make injection consistently possible although not infrequently difficult.

We confirmed a previous report that more force was required to advance a Tuohy needle tip through ligamentum flavum than through interspinous ligament^39^ and discovered that a similarly high force was required to inject fluid into supraspinous ligament.

The high-low-high pattern of serial measurements of perceived resistance to fluid injection (R_inj-f_) across the 3 lumbar dorsal midline ligamentous layers suggested that serial measurement of these resistances could reveal a pattern that could signal when the Tuohy needle tip was becoming close to the epidural space. We chose to quantify these differences by creating a subjective 10-level ordinal scale, with 1 denoting negligible R_inj-f_, such as occurs during fluid injection into subcutaneous fat, and 10 indicating complete inability to inject despite application of maximum thumb force on the syringe plunger.

The tissue waypoints to be checked for during 2- to 3-mm serial Tuohy needle insertions from skin to epidural space became the following: (a) an increase in insertion force required to advance the Tuohy needle once the needle tip, having passed through fat, contacts supraspinous ligament, (b) an increase in R_inj-f_ once the Tuohy needle end orifice becomes embedded in supraspinous ligament, (c) a reduction in R_inj-f_ when the Tuohy needle end orifice enters interspinous ligament, (d) an increase in force required to further advance the Tuohy needle when the tip encounters ligamentum flavum, (e) an increase in R_inj-f_ once the Tuohy needle end orifice becomes embedded in ligamentum flavum, and (f) a dramatic reduction in R_inj-f_ when the Tuohy needle end orifice enters the epidural space.

Two problems with using these tissue waypoints were (a) their indistinctness in some parturients, especially for novice operators, and (b) regions in some parturients’ interspinous ligaments with sufficiently low resistance to injection that cause the phenomenon of false-positive loss of resistance.^39^

A method to distinguish false-positive from true loss of resistance was recommended by a departmental colleague: after any drop in R_inj-f_ sufficient to suggest possible Tuohy needle end orifice entry into the epidural space, disconnect the 3-mL syringe from the Tuohy needle, insert the spinal needle, and then remove its stylet to check for clear cerebrospinal fluid dripping from the spinal needle hub (proving spinal needle tip entry into the subarachnoid space). This suggestion was adopted. Also, the distance the spinal needle had to be inserted beyond the tip of the Tuohy needle to penetrate dura mater and achieve spinal puncture (often accompanied by palpation of a distinctive pop) became used as a signal of how much farther the Tuohy needle might have to be advanced to permit insertion of the epidural catheter. The maximum distance the Espocan kit spinal needle can be inserted beyond the Tuohy needle tip is 15 mm.

If the spinal needle end orifice was successfully advanced into the subarachnoid space, the loading dose, or spinal dose, of local anesthetic for labor analgesia or cesarean anesthesia was injected. If the spinal needle end orifice could not be maneuvered to enter the subarachnoid space, the spinal needle was withdrawn from the Tuohy needle, the fluid-filled 3-mL syringe was reconnected, and the flush-measure-check-advance Tuohy needle insertion algorithm was recommenced.

Apart from identifying false-positive loss of R_inj-f_ in interspinous ligament, spinal needle insertion also proved useful in confirming partial Tuohy needle end orifice entry into the epidural space. After an ambiguous loss of R_inj-f_, partial Tuohy needle end orifice entry into the epidural space was inferred when a spinal needle inserted through the Tuohy needle entered the subarachnoid space, but the epidural catheter could not be inserted through it into the epidural space.

In instances when loss of R_inj-f_ was convincing for Tuohy needle entry into the epidural space but spinal needle insertion—even to full depth—did not return cerebrospinal fluid, the spinal needle was partially withdrawn, the Tuohy needle was rotated 90° to slightly alter the angle at which the spinal needle would exit the Tuohy needle tip, and the spinal needle was reinserted to again check for return of cerebrospinal fluid. This process was repeated up to 3 times before inserting the epidural catheter to perform CEA.

### Measurement of Tuohy Needle Insertion Distance Deep to Supraspinous Ligament Rather Than Skin

Three years into the study, we realized that the consistent ability to identify Tuohy needle tip entry into supraspinous ligament would permit measuring epidural space depth from this landmark rather than from skin. The depth of a patient's epidural space is the sum of (a) the distance from skin to supraspinous ligament and (b) the distance from supraspinous ligament to epidural space (Figure 2B). Thus, measuring epidural space depth from supraspinous ligament eliminates measurement variability attributable to obesity.

We therefore began to calculate real-time Tuohy needle insertion depth deep to supraspinous ligament as 9 cm (Tuohy needle shaft length), minus the number of needle cm markings still visible outside the skin, minus the depth in cm of a parturient's supraspinous ligament. Supraspinous ligament depth beneath skin was chosen to be the needle tip insertion distance at which the operator sensed the first large increase in R_inj-f_ compared to the very low R_inj-f_ of subcutaneous tissue. This first large increase in R_inj-f_ indicated the entire end orifice of the Tuohy needle was in supraspinous ligament and was consistently identifiable.

### Criterion for Diagnosis of Unintended Tuohy Needle Dural Puncture

To permit fair comparison between our rate of unintended dural puncture and previously reported rates (Figure 1), we used the traditional criterion for diagnosis of such an event: visible and usually copious flow of cerebrospinal fluid from the Tuohy needle hub after disconnection of the syringe.

### Denominators of Descriptive Statistics

Statistics related to use of the flush-measure-check-advance algorithm for Tuohy needle advancement during performance of CSEA were often based on data from fewer than the full population of 393 parturients. For example, instances of false-positive loss of R_inj-f_ began to be counted with the 20th parturient and were not always recorded thereafter. Counting Tuohy needle insertion distance from supraspinous ligament, rather than from skin, became routine starting with the 229th parturient. Also, after teaching a novice resident, the faculty anesthesiologist sometimes could not recall the depth of a parturient's supraspinous ligament or epidural space.

### Recordkeeping and Review

**Figure 3.**
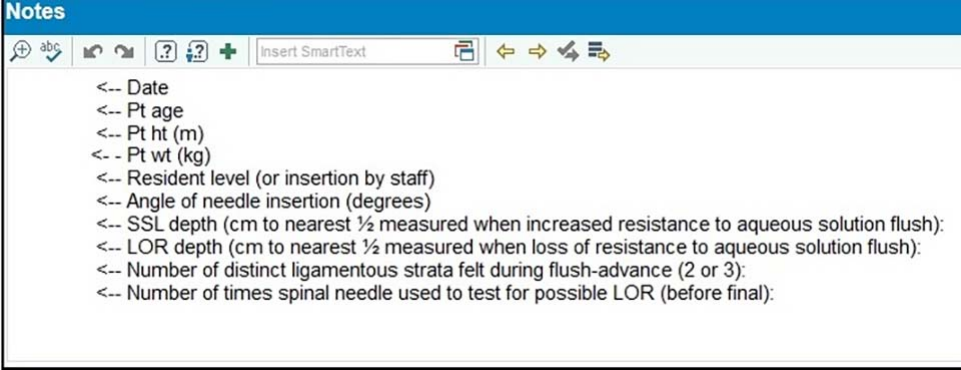
**Smart phrase addendum to the electronic medical record default combined spinal-epidural anesthesia procedure note.** ht, height; LOR, loss of resistance; Pt, patient; SSL, supraspinous ligament; wt, weight.

After initiation of CSEA, the operator performing the procedure completed an electronic medical record note. The faculty anesthesiologist later appended additional information to the note using an Epic (Epic Systems Corporation) electronic medical record smart phrase (Figure 3).

At the end of the project, the hospital system institutional review board approved a retrospective review of parturients’ neuraxial anesthesia procedure notes to permit preparation of this report.

## RESULTS

From March 2014 through January 2022, the flush-measure-check-advance technique was used in 393 parturients.

### Parturient Demographics

Parturient ages ranged from 15 to 50 years (mean 26.8 years, SD 5.6 years); heights ranged from 1.3 to 1.9 m (mean 1.6 m, SD 0.1 m); weights ranged from 40 to 191 kg (mean 85.5 kg, SD 20.0 kg); and body mass indices ranged from 17.8 to 66.1 kg/m^2^ (mean 32.7 kg/m^2^, SD 7.2 kg/m^2^).

### Neuraxial Anesthesia Success Rate

The goal of initiating local anesthetic blockade with CSEA was achieved in 373 of the 393 parturients (94.9%). In 19 parturients (4.8%), the epidural space was identified through loss of R_inj-f_, but a spinal needle inserted through the Tuohy needle failed to return cerebrospinal fluid. Consequently, only CEA was provided. In 1 parturient (0.3%), Tuohy needle use was abandoned, and neuraxial anesthesia was achieved using a spinal needle.

### Incidence of Unintended Tuohy Needle Dural Puncture

The rate of witnessed unintended Tuohy needle dural puncture was 0%. One parturient received 2 separate CSEAs because of inadequate pain relief from the first CSEA. She developed a typical post-dural puncture headache despite no visible sign of Tuohy needle dural puncture during either procedure.

### Estimating Tuohy Needle Tip Proximity to the Epidural Space by Interpreting the Serial Measurements of Resistance to Fluid Injection

An abrupt reduction in R_inj-f_ while the Tuohy needle tip was within interspinous ligament was scored as a false-positive loss of resistance if a spinal needle inserted through the Tuohy needle did not return cerebrospinal fluid but with continued Tuohy needle insertion, the Tuohy needle end orifice did eventually enter the epidural space and permitted epidural catheterization (usually after performance of spinal anesthesia).

Any reduction in R_inj-f_ that was followed by return of cerebrospinal fluid through a spinal needle inserted through the Tuohy needle was scored as a true-positive loss of resistance.

Figures 2A and 2C illustrate the relationship between depth of Tuohy needle insertion and serial measurements of R_inj-f_.

**Figure 4.**
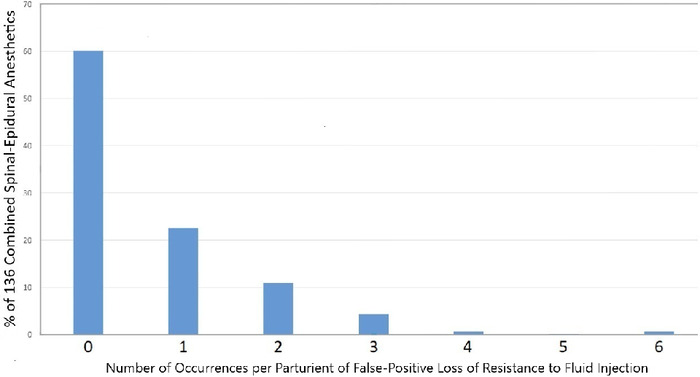
Frequency of occurrences of false-positive loss of resistance to fluid injection expressed as percentage of 136 parturients receiving combined spinal-epidural anesthesia.

The occurrence or lack of occurrence of false-positive loss of R_inj-f_ was recorded for 136 parturients. In most parturients (82/136, 60.3%), no false-positive loss of R_inj-f_ occurred. Figure 4 shows the percentages of parturients with 1 to 6 instances of false-positive loss of R_inj-f_. A single occurrence of false-positive loss of R_inj-f_ occurred in 31 of 136 parturients (22.8%). A false-positive loss of resistance is depicted in Figure 2C.

### Reduction of Variability in the Measurement of Epidural Space Depth

**Figure 5.**
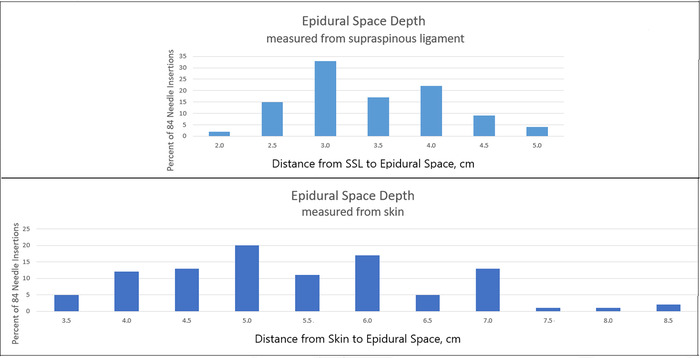
Epidural space depth measured deep to supraspinous ligament (SSL) (upper graph) and deep to skin (lower graph) in the same 84 parturients.

Figure 5 shows that the range of epidural space depth measurements was reduced by 40% (from 5 to 3 cm) when Tuohy needle insertion depth was counted from supraspinous ligament (range, 2.0-5.0 cm) rather than from skin (range, 3.5-8.5 cm). The number of parturients used to create the graphs in Figure 5 (n=84) is smaller than the number used to create the Figure 4 graph (n=136) because we began measuring the depth of the epidural space from supraspinous ligament rather than from skin 18 months after we began recording the instances of false-positive loss of R_inj-f_.

### Additional Tuohy Needle Advancements Needed to Catheterize the Epidural Space

**Figure 6.**
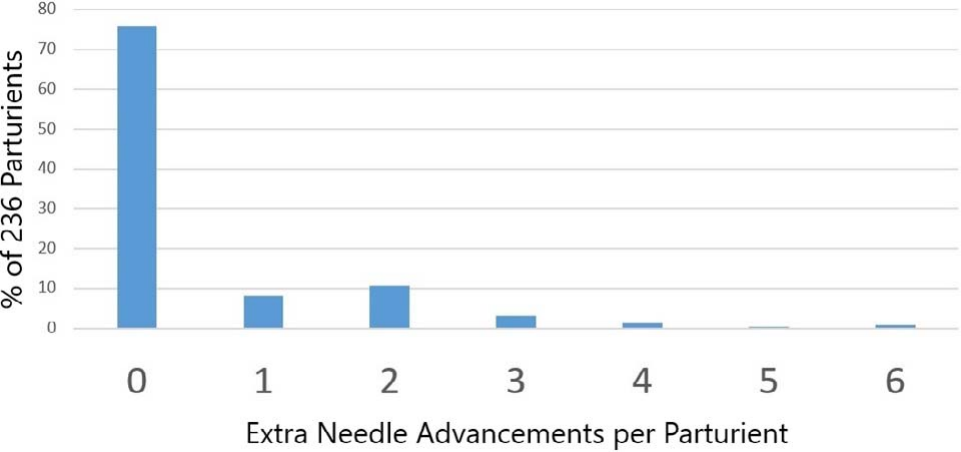
Number of 1- to 2-mm Tuohy needle advancements required to insert the catheter through the Tuohy needle into the epidural space after spinal anesthesia was performed in 236 parturients.

Figure 6 shows the number of extra 1- to 2-mm Tuohy needle advancements that were required to insert the catheter into the epidural space after spinal anesthesia was performed in 236 parturients. In 75% of parturients, no additional Tuohy needle advancements were required to permit epidural catheterization. In 1 parturient, however, 6 extra Tuohy needle advancements were needed. Usually, when 1 or more extra Tuohy needle advancements were required, R_inj-f_ after the final advance was noticeably less than for the previous one(s). For every parturient in whom spinal needle insertion through a Tuohy needle reached the subarachnoid space (n=236), epidural catheterization was achieved.

### Tuohy Needle Blockage

Although considerable force was often needed to flush fluid from the 3-mm Luer lock syringe attached to the Tuohy needle into supraspinous ligament or ligamentum flavum, on one occasion, manual injection was impossible despite application of maximum thumb force (R_inj-f_=10). In this instance, the Tuohy needle tip was withdrawn to shallow subcutaneous tissue, the entire needle was lifted cephalad approximately 1 mm, and the needle was then reinserted. Forceful insertion of the Tuohy needle stylet failed to dislodge the obstruction, so the Tuohy needle was completely withdrawn and examined: a plug of periosteal tissue had become securely lodged in the end orifice and had to be pried out using the point of an 18-gauge A-bevel needle.

### Tuohy Needle Rotation

Six parturients required Tuohy needle rotation to achieve cerebrospinal fluid flow through the spinal needle followed by injection of the spinal dose of local anesthesia. Despite a published warning not to turn the Tuohy needle once the tip was within the epidural space to avoid unintended dural puncture,^40^ this maneuver caused no dural puncture in any of our parturients.

### Physician Experience

Physician experience level was recorded for 384 parturients. Sixty-nine percent (265/384) of procedures were performed by the faculty member, 13.5% (52/384) by clinical year 2 or 3 residents, and 17.4% (67/384) by clinical year 1 residents (novices).

## DISCUSSION

In our quality improvement clinical series that involved 393 parturients, use of the flush-measure-check-advance algorithm to perform CSEA was associated with a 0% rate of witnessed unintended dural puncture. This rate is low considering that 17.4% of the procedures were performed by novices whose expected unintended dural puncture rate is approximately 3%.^37^

### Single Occurrence of Post-Dural Puncture Headache

One parturient who required 2 separate CSEAs experienced a post-dural puncture headache despite no evidence of Tuohy needle dural puncture. Considering that the rate of post-dural puncture headache in parturients who receive spinal anesthesia through a 25-gauge pencil-point needle is approximately 2%,^41^ this parturient's headache could be attributable to intended spinal needle dural puncture. Eley et al have proposed classifying the causative agent of any post-dural puncture headache occurring without visible evidence of Tuohy needle entry into the subarachnoid space as an “unrecognized dural puncture.”^42^ If our parturient's postpartum headache is attributed to an unrecognized dural puncture, the rate of unintended Tuohy needle dural puncture in our series is 0.25% (1/393).

### Causes of Unintended Tuohy Needle Dural Puncture

Multiple factors contribute to unintended Tuohy needle dural puncture during neuraxial anesthesia, including operator inexperience, lack of recent practice, low quality supervision of novice physicians, difficult patient anatomy (obesity, edema, lumbar lordosis, deep epidural space, scoliosis), and tissue blockage of the Tuohy needle end orifice that prevents recognition of the needle entry into the epidural space.^37,38,43^

Assuming the flush-measure-check-advance Tuohy needle insertion algorithm can consistently help prevent unintended Tuohy needle dural puncture, our data are insufficient to establish the relative importance of the individual elements: (a) flushing saline or local anesthetic solution through the Tuohy needle after each advancement to clear the needle tip of any obstructing tissue, (b) using tissue resistance to needle advancement and R_inj-f_ to improve tracking Tuohy needle tip position within the 3 lumbar midline ligamentous layers and to warn of tip proximity to the epidural space, and (c) after detecting loss of resistance to fluid injection sufficient to suggest possible Tuohy needle tip entry into this space, inserting a spinal needle through the Tuohy needle to confirm the presence of cerebrospinal fluid just ahead of it.

### Limitations

While this quality improvement clinical series describes what we consider a noteworthy decrease in the number of unintended dural punctures during the performance of neuraxial anesthesia and analgesia in parturients by resident trainees, specific limitations must be acknowledged. Our quality improvement project includes a relatively small sample size at a single institution and is retrospective in nature. Furthermore, data collection was subject to inconsistencies during the evolution of the project, resulting in descriptive statistics based on fewer than the 393 total number of parturients.

## CONCLUSION

A faculty anesthesiologist and supervised residents achieved a 0% rate of witnessed unintended Tuohy needle dural puncture during the performance of CSEA in 373 parturients and CEA in 19 parturients using a Tuohy needle insertion algorithm that included traditional and new elements. Because our quality improvement clinical series was neither large nor a randomized controlled study, the ability of flush-measure-check-advance technique to prevent unintended Tuohy needle puncture of the dura mater and post-dural puncture headache in parturients requires confirmation.

## Notes

*This article meets the Accreditation Council for Graduate Medical Education and the American Board of Medical Specialties Maintenance of Certification competencies for Patient Care, Medical Knowledge, and Practice-Based Learning and Improvement.*

## References

[1] Committee on Obstetric Anesthesia. Statement on post-dural puncture headache management. American Society of Anesthesiologists. October 13, 2021. Accessed May 8, 2025. asahq.org/standards-and-practice-parameters/statement-on-post-dural-puncture-headache-management

[2] Crawford JS. Lumbar epidural block in labour: a clinical analysis. Br J Anaesth. 1972;44(1):66-74. doi: 10.1093/bja/44.1.66 5058136

[3] Kalas DB, Hehre FW. Continuous lumbar peridural anesthesia in obstetrics. 8. Further observations on inadvertent lumbar puncture. Anesth Analg. 1972;51(2):192-195. 5062117

[4] Craft JB, Epstein BS, Coakley CS. Prophylaxis of dural-puncture headache with epidural saline. Anesth Analg. 1973;52(2):228-231. 4735287

[5] Brownridge P. The management of headache following accidental dural puncture in obstetric patients. Anaesth Intensive Care. 1983;11(1):4-15. doi: 10.1177/0310057X8301100102 6859506

[6] Okell RW, Sprigge JS. Unintentional dural puncture. A survey of recognition and management. Anaesthesia. 1987;42(10):1110-1113. doi: 10.1111/j.1365-2044.1987.tb05181.x 3688397

[7] Norris MC, Leighton BL, DeSimone CA. Needle bevel direction and headache after inadvertent dural puncture. Anesthesiology. 1989;70(5):729-731. doi: 10.1097/00000542-198905000-00002 2655500

[8] MacArthur C, Lewis M, Knox EG. Accidental dural puncture in obstetric patients and long term symptoms. BMJ. 1993;306(6882):883-885. doi: 10.1136/bmj.306.6882.883 8490410 PMC1677341

[9] Stride PC, Cooper GM. Dural taps revisited. A 20-year survey from Birmingham Maternity Hospital. Anaesthesia. 1993;48(3):247-255. doi: 10.1111/j.1365-2044.1993.tb06913.x 8460807

[10] Trivedi NS, Eddi D, Shevde K. Headache prevention following accidental dural puncture in obstetric patients. J Clin Anesth. 1993;5(1):42-45. doi: 10.1016/0952-8180(93)90086-t 8442966

[11] Norris MC, Grieco WM, Borkowski M, Complications of labor analgesia: epidural versus combined spinal epidural techniques [published correction appears in *Anesth Analg* 1994 Dec;79(6):1217]. Anesth Analg. 1994;79(3):529-537. doi: 10.1213/00000539-199409000-00022 8067559

[12] Palot M, Visseaux H, Botmans C, Pire JC. Epidemiology of complications of obstetrical epidural analgesia. Article in French. Cah Anesthesiol. 1994;42(2):229-233. 8087639

[13] Collis RE, Davies DW, Aveling W. Randomised comparison of combined spinal-epidural and standard epidural analgesia in labour. Lancet. 1995;345(8962):1413-1416. doi: 10.1016/s0140-6736(95)92602-x 7760614

[14] Paech MJ, Godkin R, Webster S. Complications of obstetric epidural analgesia and anaesthesia: a prospective analysis of 10,995 cases. Int J Obstet Anesth. 1998;7(1):5-11. doi: 10.1016/s0959-289x(98)80021-6 15321239

[15] Berger CW, Crosby ET, Grodecki W. North American survey of the management of dural puncture occurring during labour epidural analgesia. Can J Anaesth. 1998;45(2):110-114. doi: 10.1007/BF03013247 9512843

[16] Huffnagle SL, Norris MC, Arkoosh VA, The influence of epidural needle bevel orientation on spread of sensory blockade in the laboring parturient. Anesth Analg. 1998;87(2):326-330. doi: 10.1097/00000539-199808000-00017 9706925

[17] Gleeson CM, Reynolds F. Accidental dural puncture rates in UK obstetric practice. Int J Obstet Anesth. 1998;7(4):242-246. doi: 10.1016/s0959-289x(98)80046-0 15321187

[18] Richardson MG, Wissler RN. The effects of needle bevel orientation during epidural catheter insertion in laboring parturients. Anesth Analg. 1999;88(2):352-356. doi: 10.1097/00000539-199902000-00024 9972755

[19] Rutter SV, Shields F, Broadbent CR, Popat M, Russell R. Management of accidental dural puncture in labour with intrathecal catheters: an analysis of 10 years' experience. Int J Obstet Anesth. 2001;10(3):177-181. doi: 10.1054/ijoa.2001.0854 15321607

[20] Comparative Obstetric Mobile Epidural Trial (COMET) Study Group UK. Effect of low-dose mobile versus traditional epidural techniques on mode of delivery: a randomised controlled trial. Lancet. 2001;358(9275):19-23. doi: 10.1016/S0140-6736(00)05251-X 11454372

[21] Paech M, Banks S, Gurrin L. An audit of accidental dural puncture during epidural insertion of a Tuohy needle in obstetric patients. Int J Obstet Anesth. 2001;10(3):162-167. doi: 10.1054/ijoa.2000.0825 15321604

[22] Norris MC, Fogel ST, Conway-Long C. Combined spinal-epidural versus epidural labor analgesia. Anesthesiology. 2001;95(4):913-920. doi: 10.1097/00000542-200110000-00020 11605932

[23] van de Velde M, Teunkens A, Hanssens M, van Assche FA, Vandermeersch E. Post dural puncture headache following combined spinal epidural or epidural anaesthesia in obstetric patients. Anaesth Intensive Care. 2001;29(6):595-599. doi: 10.1177/0310057X0102900605 11771601

[24] Pan PH, Bogard TD, Owen MD. Incidence and characteristics of failures in obstetric neuraxial analgesia and anesthesia: a retrospective analysis of 19,259 deliveries. Int J Obstet Anesth. 2004;13(4):227-233. doi: 10.1016/j.ijoa.2004.04.008 15477051

[25] Evron S, Sessler D, Sadan O, Boaz M, Glezerman M, Ezri T. Identification of the epidural space: loss of resistance with air, lidocaine, or the combination of air and lidocaine. Anesth Analg. 2004;99(1):245-250. doi: 10.1213/01.ANE.0000120084.56136.15 15281538

[26] De Blas García M, Guasch Arévalo E, Martínez Jiménez F, Gredilla Díez E, Gilsanz Rodríguez F. Analysis of resident anesthesiologists' difficulties with epidural analgesia for labor and childbirth and complication rates. Article in Spanish. Rev Esp Anestesiol Reanim. 2007;54(2):78-85. 17390689

[27] Sprigge JS, Harper SJ. Accidental dural puncture and post dural puncture headache in obstetric anaesthesia: presentation and management: a 23-year survey in a district general hospital. Anaesthesia. 2008;63(1):36-43. doi: 10.1111/j.1365-2044.2007.05285.x 18086069

[28] Van de Velde M, Schepers R, Berends N, Vandermeersch E, De Buck F. Ten years of experience with accidental dural puncture and post-dural puncture headache in a tertiary obstetric anaesthesia department. Int J Obstet Anesth. 2008;17(4):329-335. doi: 10.1016/j.ijoa.2007.04.009 18691871

[29] Katircioglu K, Hasegeli L, Ibrahimhakkioglu HF, Ulusoy B, Damar H. A retrospective review of 34,109 epidural anesthetics for obstetric and gynecologic procedures at a single private hospital in Turkey. Anesth Analg. 2008;107(5):1742-1745. doi: 10.1213/ane.0b013e31817bd11f 18931241

[30] Singh S, Chaudry SY, Phelps AL, Vallejo MC. A 5-year audit of accidental dural punctures, postdural puncture headaches, and failed regional anesthetics at a tertiary-care medical center. ScientificWorldJournal. 2009;9:715-722. doi: 10.1100/tsw.2009.94 19649510 PMC5823092

[31] Darvish B, Gupta A, Alahuhta S, Management of accidental dural puncture and post-dural puncture headache after labour: a Nordic survey. Acta Anaesthesiol Scand. 2011;55(1):46-53. doi: 10.1111/j.1399-6576.2010.02335.x 21039355

[32] Bellas S, Marenco ML, Sepúlveda A, Suan C. Incidence of accidental dura mater punctures in a university hospital: a prospective observational study. Article in Spanish. Rev Esp Anestesiol Reanim. 2012;59(8):410-414. doi: 10.1016/j.redar.2012.03.023 22609267

[33] Deighan M, Briain DO, Shakeban H, A randomised controlled trial using the Epidrum for labour epidurals. Ir Med J. 2015;108(3):73-75. 25876297

[34] Peralta F, Higgins N, Lange E, Wong CA, McCarthy RJ. The relationship of body mass index with the incidence of postdural puncture headache in parturients. Anesth Analg. 2015;121(2):451-456. doi: 10.1213/ANE.0000000000000802 25993388

[35] Antunes MV, Moreira A, Sampaio C, Faria A. Accidental dural puncture and post-dural puncture headache in the obstetric population: eight years of experience. Article in Portuguese. Acta Med Port. 2016;29(4):268-274. doi: 10.20344/amp.6815 27349779

[36] Guglielminotti J, Landau R, Li G. Major neurologic complications associated with postdural puncture headache in obstetrics: a retrospective cohort study. Anesth Analg. 2019;129(5):1328-1336. doi: 10.1213/ANE.0000000000004336 31335402 PMC9924132

[37] Sidhu NS, Cavadino A, Ku H, Kerckhoffs P, Lowe M. The association between labour epidural case volume and the rate of accidental dural puncture. Anaesthesia. 2021;76(8):1060-1067. doi: 10.1111/anae.15370 33492698

[38] Cohen S, Maestrado P, Zada Y. A simple technique to reduce the incidence of accidental dural puncture. Anaesthesia. 2001;56(7):708. doi: 10.1046/j.1365-2044.2001.02137-28.x 11463040

[39] Sharrock NE. Recordings of, and an anatomical explanation for, false positive loss of resistance during lumbar extradural analgesia. Br J Anaesth. 1979;51(3):253-258. doi: 10.1093/bja/51.3.253 435350

[40] Duffy BL. “Don't turn the needle!” Anaesth Intensive Care. 1993;21(3):328-330. doi: 10.1177/0310057X9302100312 8342763

[41] Maranhao B, Liu M, Palanisamy A, Monks DT, Singh PM. The association between post-dural puncture headache and needle type during spinal anaesthesia: a systematic review and network meta-analysis. Anaesthesia. 2021;76(8):1098-1110. doi: 10.1111/anae.15320 33332606

[42] Eley VA, Abeypala W, Kelley A, Kumta N, Chin A. Recognized and unrecognized dural punctures in 12,981 labor epidurals: an audit of management. J Anesth. 2022;36(3):399-404. doi: 10.1007/s00540-022-03062-7 35474399 PMC9156467

[43] Kakde A, Chia P, Tan HS, Sultana R, Tan CW, Sng BL. Factors associated with an inadvertent dural puncture or post-dural puncture headache following labour epidural analgesia: a retrospective cohort study. Heliyon. 2024;10(6):e27511. doi: 10.1016/j.heliyon.2024.e27511 38501002 PMC10945181

